# JQ1 as a BRD4 Inhibitor Blocks Inflammatory Pyroptosis-Related Acute Colon Injury Induced by LPS

**DOI:** 10.3389/fimmu.2021.609319

**Published:** 2021-02-18

**Authors:** Ling Chen, Xiaolin Zhong, Wenyu Cao, Mingli Mao, Wei Li, Hui Yang, Menglin Li, Mengmeng Shi, Yuan Zhang, Yincheng Deng, Xuyu Zu, Jianghua Liu

**Affiliations:** ^1^ Department of Endocrinology and Metabolism, The First Affiliated Hospital of University of South China, Hengyang, China; ^2^ Clinical Anatomy & Reproductive Medicine Application Institute, Hengyang Medical School, University of South China, Hengyang, China; ^3^ Department of Pathology, Hengyang Medical School, University of South China, Hengyang, China; ^4^ Department of Tumor Research, The First Affiliated Hospital of University of South China, Hengyang, China

**Keywords:** colon injury, BRD4, pyroptosis, JQ1, endotoxemia****

## Abstract

Endotoxemia is a severe inflammation response induced by infection especially bacterial endotoxin translocation, which severely increases mortality in combination with acute colon injury. Bromodomain-containing protein 4 (BRD4) is an important Bromo and Extra-Terminal (BET) protein to participate in inflammatory responses. However, it is still unknown about the specific connection between BRD4 and inflammation-related pyroptosis in endotoxemia colon. Here, through evaluating the mucous morphology and the expression of tight junction proteins such as occludin and ZO1, we found the upregulation of BRD4 in damaged colon with poor tight junction in an endotoxemia mouse model induced by lipopolysaccharides (LPS). Firstly, the BRD4 inhibitor JQ1 was used to effectively protect colon tight junction in endotoxemia. As detected, high levels of pro-inflammation cytokines IL6, IL1β and IL18 in endotoxemia colon were reversed by JQ1 pretreatment. In addition, JQ1 injection reduced endotoxemia-induced elevation of the phosphorylated NF κB and NLRP3/ASC/caspase 1 inflammasome complex in colon injury. Furthermore, activated pyroptosis markers gasdermins in endotoxemia colon were also blocked by JQ1 pretreatment. Together, our data indicate that BRD4 plays a critical role in regulating pyroptosis-related colon injury induced by LPS, and JQ1 as a BRD4 inhibitors can effectively protect colon from endotoxemia-induced inflammation injury.

## Introduction

Bacterial endotoxin translocation from gastrointestinal tract to the systemic circulation to cause endotoxemia (1). As an acute infection-related inflammatory response, endotoxemia is reported to exacerbate atherosclerosis, steatosis, insulin resistance, and atherosclerosis, and to cause various biological and clinical effects in the host (1, 2). And lipopolysaccharides (LPS) is a typical bacterial endotoxin to extensively applied in bacterial endotoxemia mice model (3). Most endotoxemia-related gut disease study is mainly focused on the intestine, especially jejunal (4–6). However, compared with endotoxemia-related intestine injury, there are relatively less study on endotoxemia-induced colon injury, which is also a common complication of endotoxemia. Colon plays a key role in human immune system with distinct microbial and immune niches (7). It is meaningful to explore the special function of colon in the inflammatory response of endotoxemia. Herein, our study focuses on the endotoxemia-related colon barrier dysfunction, especially the tight junction damage.

Bromodomain-containing protein 4 (BRD4) is an important member of the Bromo and Extra-Terminal (BET) family, the bromodomains of which may bind to acetylated histones and transcription factors to regulate multiple pathophysiological activities including cancer and inflammation (8–10). As a cell-permeable small molecule, (+)-JQ1 (JQ1) reversibly and competitively binds to bromodomains to further disturb BRD4 activity (11). As a BRD4 inhibitor, JQ1 is firstly discovered as an anti-tumor compounds (12, 13), but growing evidences indicate that JQ1 becomes a recognized anti-inflammatory tool to study the functions of BRD4 particularly in inflammation-related diseases, such as cardiac hypertrophy and periodontitis (14–16). Only several studies refer to the connection between BRD4 and acute injury. For example, it is reported that lncRNA XIST silencing protects against sepsis-induced acute liver injury *via* inhibition of the BRD4 expression (17), and endoplasmic reticulum stress can suppress hepatic molecular identity through decommissioning of BRD4 super-enhancers in damaged liver (18). However, there is no study concerns about the effect of BRD4 inhibition on endotoxemia-related colon damage. In this study, we firstly use the effective inhibitor JQ1 to explore how BRD4 functions in acute colon injury in endotoxemia.

Pyroptosis is a novel type of inflammatory programmed cell death. Activated NLRP3 binds to apoptosis-associated speck-like protein (ASC) containing a caspase activation and recruitment domain, and then caspase 1 is recruited together to assemble an inflammasome complex, which finally triggers caspase 1-dependent release of the pro-inflammatory cytokines IL1β and IL18, and induces gasdermins-mediated pyroptotic cell death (19–21). Gasdermins which are responsible for cell pore-forming, such as gasdermin D (GSDMD), gasdermin E (GSDME), and gasdermin A (GSDMA), are now recognized as the gold standard markers of pyroptosis (22–24). It is reported that pyroptosis inhibition by impeding the function of GSDMD N-terminal can prevent lipopolysaccharide (LPS)-induced lethal septic shock (25, 26). So far, only a few relevant reports as we know that show the possible connection between BRD4 and pyroptosis, which mainly exists in cerebral ischemia-induced brain damage and the progress of renal cell carcinoma (27, 28). However, the correlation between BRD4 and pyroptosis in endotoxemia colon remains elusive.

In the present study, we found that BRD4 expression was upregulated in colon of LPS-induced endotoxemia mice. The BRD4 inhibitor JQ1 blocked LPS-induced colon injury by recuperative tight junction, reducing inflammation and pyroptosis.

## Materials and Methods

### Animals

Nine- to 10-week-old male C57BL/6 mice (Silaikejingda, Changsha, China) were utilized for all experiments. Mice were maintained on a 12 h light-dark cycle and treated with standard laboratory mouse chow and water *ad libitum*. All experiments were performed in accordance with the National Institutes of Health Guidelines for the Use of Laboratory Animals and were approved by the Institutional Animal Care and Use Committee at the First Affiliated Hospital of University of South China.

### LPS-Induced Endotoxemia Mice Model

Acute colon injury mice model was built by intraperitoneal injection (i.p.) with 10 mg/kg LPS (Sigma, Shanghai, China) for 24 h as described before (29–31). BRD4 inhibition was applied by i.p. pretreatment with 50 mg/kg JQ1 (MCE, Shanghai, China) for 1 h before LPS injection. The control group were intraperitoneally treated with normal saline or DMSO. Then, mice were anesthetized to obtain peripheral blood by eyeball removal. After taking out the colon tissues, longitudinally cut and wash colons with PBS. Divide colon into 0.5 cm segments, one of which was fixed in 4% paraformaldehyde, and others were stored at −80°C for further Q-PCR and western blot.

### HE Staining and Pathology Scores

After fixation in 4% paraformaldehyde overnight, the colon tissues were routinely dehydrated, transparentized, waxed, and paraffin embedded. Paraffin blocks were cut into thin sections with a thickness of 5 µm. Then the sections were deparaffinized, hydrated, and stained with hematoxylin and eosin solution. HE images were obtained randomly from the stained colon sections. Pathology scores were based on colon HE staining. In brief, the scoring system shows as below, epithelium: normal morphology (0), loss of goblet cells (1), loss of goblet cells in large areas (2), loss of crypts (3), and loss of crypts in large areas (4); infiltration: no infiltrate (0), infiltrate around crypts (1), infiltrate into the lamina muscularis mucosae (2), extensive infiltration into the lamina muscularis mucosae and thickening of the mucosa (3), and infiltration of the submucosal layer (4). The total histological score for each HE-stained section ranges from 0 to 8.

### RNA Extraction and Q-PCR

Total RNA was extracted from colon tissues using the ultra-pure RNA quick extraction kit (CWBIO, Beijing, China), and reversely transcribed to cDNA by the RevertAid First Strand cDNA Synthesis Kit (ThermoFisher, Shanghai, China). The real-time polymerase chain reaction (PCR) kit with SYBR Green dyes (Takara, Shanghai, China) was used to test the mRNA expression. The forward and reverse primers of target mRNAs were bought from the primer-synthesis company (Sangon, Shanghai, China). Sequences of primers were listed in **Table 1**.

**Table 1:** Information of primer sequences.

Gene	Sense	Anti-sense
IL6	CTCCCAACAGACCTGTCTATAC	CCATTGCACAACTCTTTTCTCA
IL18	CTGTTGGCCCAATTACTAACAG	TCCCGAATTGGAAAGGGAAATA
IL1β	GCAGAGCACAAGCCTGTCTTCC	ACCTGTCTTGGCCGAGGACTAAG
NLRP3	CGTTGCAAGCTGGCTCAGTA	GGGGACTGGGATACAGCCTT
ASC	ACAATGACTGTGCTTAGAGACA	CACAGCTCCAGACTCTTCTTTA
caspase1	AGAGGATTTCTTAACGGATGCA	TCACAAGACCAGGCATATTCTT
GSDMD	CTAGCTAAGGCTCTGGAGACAA	GATTCTTTTCATCCCAGCAGTC
GSDME	GAGAGTCACTCTTCGTTTGGAA	CTGAAGTACCAGGTTGTCCATA
GSDMA	GAACTTGCACAAGGAGAGGAAA	CATCACCACATAGAGGTTCTCC
GAPDH	ACCACCATGGAGAAGGCTGG	CTCAGTGTAGCCCAGGATGC

### Western Blot

The colon tissues were lysed in tissue lysis buffer (50 mM Tris-HCl pH 7.4, 150 mM NaCl, 1 mM PMSF, 1 mM EDTA, 5 µg/ml Aprotinin, 5 µg/ml Leupeptin, 1% Triton X-100, 1% Sodium deoxycholate, and 0.1% SDS) with a tissue homogenizer (Jingxin, Shanghai, China). Protein solution was separated by centrifuge for 30 min at 4°C, and then denatured for 10 min at 100°C. After SDS-PAGE and transmembrane process, membranes were incubated with primary antibodies specific for the following proteins: BRD4 (Abclonal, A2249), occludin (Proteintech, 27260-1-AP), ZO1 (Proteintech, 21773-1-AP), IL6 (Proteintech, 21865-1-AP), IL18 (Proteintech, 10663-1-AP), IL1β (Proteintech, 16806-1-AP), p-NF κB (CST, 3033), NF κB (CST, 8242S), NLRP3 (CST, 15101S), ASC (Santan Cruze, sc-514414), cleaved-caspase 1 (Santan Cruze, sc56036), GSDMD (Abclonal, A18281), cleaved-N-terminal GSDME (Abcam, ab222407), GSDMA (Bioss, bs-16331R). Anti-β-actin antibody (Proteintech, 60004-1-Ig) was used as a loading control.

### Immunofluorescence

Dewaxed and rehydrated slides of colon sections were boiled in citrate-EDTA antigen retrieval solution for 10 min, and were blocked with 10% normal goat serum. And then incubated with rabbit occludin primary antibody (Proteintech, 27260-1-AP, 1:200) or mouse BRD4 primary antibody (Proteintech, 67374-1-Ig, 1:100) overnight at 4°C followed by Alexa Fluor 488-Affinipure Goat Anti-Rabbit IgG (Jackson, JAC-111-545-144, 1:1,000) or Alexa Fluor 594-Affinipure Goat Anti-Mouse IgG (Jackson, JAC-111-545-144, 1:1,000). 4’,6-dia-midino-2-phenylindole (DAPI) was used for DNA counterstain.

### Colonic Permeability Assay

Two hundred microliters FITC-dextran (Sigma, 40 mg/kg) was injected into 5 cm colon pouches, and the colons are incubated in 5 ml 0.9% saline for 30 min at 37°C. Then, 150 μl saline is transferred to detect the OD value at 490 nm. FITC-dextran contents in saline are calculated according to the standard curve, and the FITC-dextran permeation rate is used to represent the injury degree of colonic epithelial barrier.

### Statistical Analysis

All results were analyzed with GraphPad Prism 5.01 software. Two-tailed Student’s t-test was applied to the comparison between the two groups. Others were analyzed with one-way analysis of variance (One-way ANOVA). The statistically significant differences were shown, and differences at *p* < 0.05 were consider significant. The data are presented as the means ± standard deviation (SD).

## Results

### Endotoxemia Induced Tight Junction Barrier Dysfunction in Mice Colon

**Figure 1:**
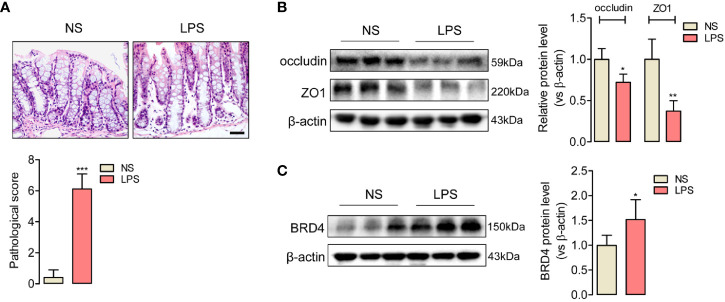
Upregulated expression of BRD4 in endotoxemia mice colon with tight junction barrier injury. **(A)** HE staining and relative pathological scores of colon tissues from control mice (NS, normal saline, i.p.), and endotoxemia mice which were treated with LPS for 24 h (10 mg/kg, i.p.). Magnification (400×) bar represents 50 μm in all panels. **(B)** Western blot showed the reduced expression of tight junction protein occludin and ZO1 in colon of endotoxemia mice compared to saline-treated mice. **(C)** BRD4 protein level was upregulated in colon of endotoxemia mice. ^*^ *p* < 0.05, ^**^ *p* < 0.01, n = 5/group, two-tailed unpaired Student’s t-test.

To evaluate the tight junction function of colon during endotoxemia, we used an endotoxemia mice model induced by intraperitoneal injection with the lipopolysaccharide (LPS, 10 mg/kg), and then obtained the images of HE-staining sections of the colonic mucosa. Disordered colon mucosa structure with crypt atrophy and goblet cell reduction were found in endotoxemia mice (**Figure 1A**). Q-PCR results of markers of Paneth cells, goblet cells, stem cells, lymphocytes, T cells, and microphages (**Figure S1**). Colonic tight junction barriers were evaluated by the expression of tight junction proteins occludin and ZO1. Lower protein levels of occludin and ZO1 in endotoxemia colons compared to the saline-treated control were detected by western blot (**Figure 1B**). Our results confirmed that endotoxemia induced severe colonic tight junction barrier dysfunction with lower expression of occludin and ZO1.

### JQ1 as a Novel BRD4 Inhibitor Prevented Endotoxemia-Induced Colonic Tight Junction Barrier Dysfunction

**Figure 2:**
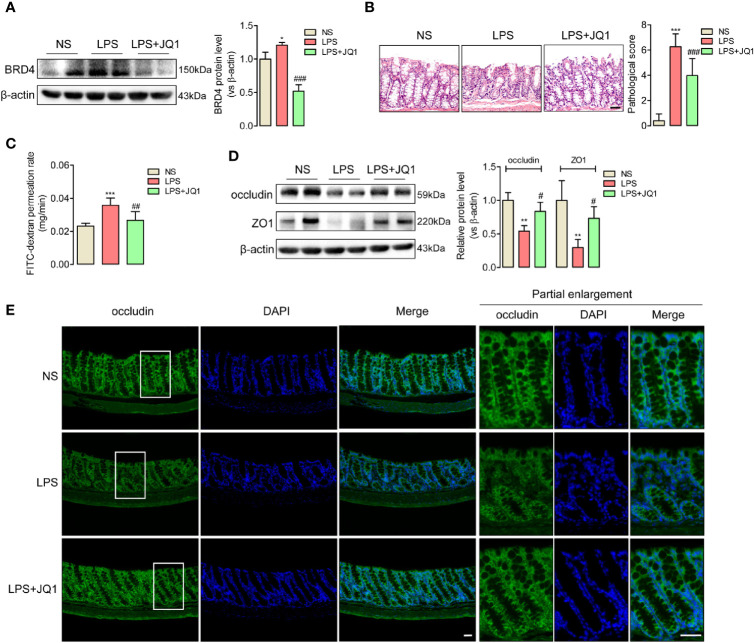
JQ1 as a novel BRD4 inhibitor repaired the tight junction damage of endotoxemia colon. Mice were injected with JQ1 (50 mg/kg, i.p.) 1 h before LPS (10 mg/kg) injection. NS group was treated with DMSO, which was the solvent of JQ1. Colon were harvested 24 h after LPS injection for western blot, HE staining, and IF assay. **(A)** JQ1 blocked BRD4 protein level in colon of endotoxemia mice. **(B)** HE staining and relative pathological scores showed pretreatment with BRD4 inhibitor JQ1 reversed the injury of colon mucosal layer. 400×, bar value was 50 μm. **(C)** Colonic permeability assay indicated the protection function of JQ1 in colonic barriers. **(D)** Expression of tight junction protein occludin and ZO1 in endotoxemia colon were detected by western blot. **(E)** Immunofluorescence of occludin also indicated the protection role of JQ1 in endotoxemia colon. Bar value was 50 μm. ^*^ *p* < 0.05, ^**^ *p* < 0.01, ***p < 0.001, *vs* NS group. ^#^ *p* < 0.05, ^##^ *p* < 0.01, ^###^ *p* < 0.001, *vs* LPS group. n = 5/group, one-way ANOVA.

BRD4 worked as a vital pro-inflammation factor in inflammation-related diseases such as osteoarthritis and vascular inflammation (32, 33). In order to explore whether BRD4 also play a specific role in endotoxemia, western blot assay was used to detect the protein changes of BRD4 in endotoxemia colons compared to control colons. It was interesting that BRD4 protein level obviously increased in endotoxemia colons (**Figure 1C**). Immunofluorescence co-localization confirmed the expression of BRD4 in colon (**Figure S2**). Here, we used JQ1, a novel and widely used compound for BET bromodomain inhibition of BRD4 (11), to further evaluate how BRD4 functioned in endotoxemia colon. The protein expression of BRD4 in endotoxemia colons was powerfully inhibited by pretreatment with JQ1 (50 mg/kg, i.p.) (**Figure 2A**). HE staining showed pretreatment with JQ1 attenuated the severity degree of endotoxemia-induced colonic mucosa damage (**Figure 2B**). High FITC-dextran permeation rate of endotoxemia colon was reduced by JQ1 (**Figure 2C**). Results of western blot (**Figure 2D**) and immunofluorescence (**Figure 2E**) disclosed that reduction of tight junction proteins in endotoxemia colon was also reversed by JQ1 treatment. These data revealed that BRD4 inhibition could block colonic tight junction barrier dysfunction induced by LPS.

### BRD4 Inhibition by JQ1 Suppressed Inflammatory Response in Endotoxemia Colon

**Figure 3:**
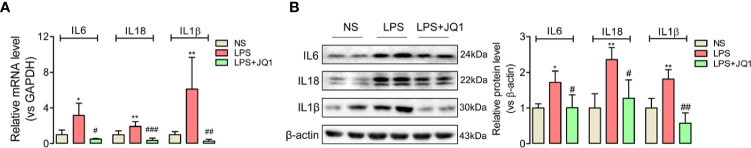
BRD4 inhibition by JQ1 blocked colon inflammation during endotoxemia. Results of Q-PCR **(A)** and western blot **(B)** showed the BRD4 inhibitor JQ1 could block the rising trend of inflammation cytokines IL6, IL18, and IL1β at gene level and protein level respectively. ^*^ *p* < 0.05, ^**^ *p* < 0.01, *vs* NS group. ^#^ *p* < 0.05, ^##^ *p* < 0.01, 0.01,^ ###^ *p* < 0.001, *vs* LPS group. n = 6/group, one-way ANOVA.

Since endotoxemia is an infection-induced systemic inflammatory response, the expressions of pro-inflammatory factors IL6, IL18, and IL1β in endotoxemia colon were calculated by Q-PCR (**Figure 3A**) and western blot (**Figure 3B**). Gene and protein expressions of the inflammatory cytokines above showed significantly accumulated trends in damaged colon, but were reduced by the pretreatment of BRD4 inhibitor JQ1 (**Figures 3B** **)**. And the anti-inflammatory factors (**Figure S3**) showed upregulation trends with JQ1 treatment. These results disclosed the potential anti-inflammation effect of BRD4 inhibitor JQ1 in endotoxemia colon.

### BRD4 Inhibition Attenuated Phosphorylated NF-κB and Blocked the Activation of NLRP3/ASC/Caspase 1 Inflammasome Complex in Endotoxemia Colon

**Figure 4:**
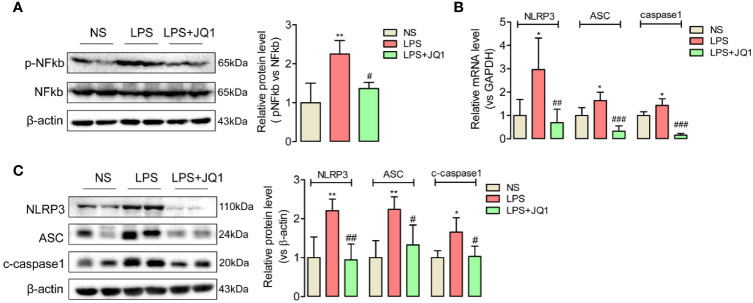
BRD4 activated phosphorylated NF κb and promoted the expression of NLRP3/ASC/Caspase 1 inflammasome complex in endotoxemia colon. **(A)** Western blot disclosed that phosphorylated NF κb was activated in endotoxemia colon, which was reversed by BRD4 inhibitor JQ1. **(B, C)** Pretreatment with JQ1 prevents gene and protein expression of the NLRP3/ASC/caspase1 inflammasome complex in endotoxemia colon. ^*^ *p* < 0.05, ^**^ *p* < 0.01, *vs* NS group. ^#^ *p* < 0.05, ^##^ *p* < 0.01, ^###^ *p* < 0.001 *vs* LPS group. n = 6/group, one-way ANOVA.

BRD4 is recognized as an emerging actor in NF-κB signaling-related inflammation (34). Because of the suppression effects of JQ1 on inflammation cytokines IL6, IL18, and IL1β in endotoxemia colons, we further confirmed effects of BRD4 inhibition on the phosphorylation of NF-κB by western blot, and found the upregulated expression of phosphorylated NF κB in endotoxemia colon was obviously reduced by JQ1 pretreatment (**Figure 4A**). Moreover, we discovered the rising gene and protein expression of an inflammasome complex containing NLRP3, ASC, and cleaved caspase 1 in endotoxemia colons compared to the control ones, which was also decreased by BRD4 inhibition (**Figures 4B, C** **)**. Data above showed that JQ1 prevented endotoxemia-related inflammation in colon through disturbing the phosphorylation of NF-κB and the activation of NLRP3/ASC/Caspase 1.

### JQ1 Inhibited BRD4 to Eliminate Endotoxemia-Induced Inflammation-Related Pyroptosis in Mice Colon

**Figure 5:**
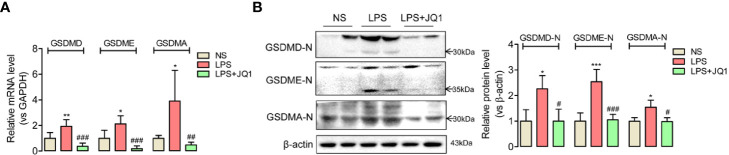
Inhibition of BRD4 blocked the expression of pyroptosis-related proteins in endotoxemia colon. In endotoxemia colon, pyroptosis-related proteins GSDMD, GSDME, and GSDMA with active forms were upregulated at gene **(A)** and protein level **(B)**, which were powerfully reversed by BRD4 inhibitor JQ1. ^*^ *p* < 0.05, ^**^ *p* < 0.01, ^***^ *p* < 0.001 *vs* NS group. ^#^ *p* < 0.05, ^##^ *p* < 0.01, ^###^ *p* < 0.001 *vs* LPS group. n = 6/group, one-way ANOVA.

Both the accumulation of inflammation cytokines especially IL18 and IL1β, and the activation of NLRP3/ASC/Caspase 1 inflammasome complex in damaged colon indicated that endotoxemia-induced colonic mucosa injury might be related with inflammation-related pyroptosis. To confirm the hypothesis, we used Q-PCR and western blot to detect the colonic expression of admitted pyroptosis markers, the gasdermin family such as GSDMD, GSDME, and GSDMA. The active-formed gasdermins showed obvious upregulated trends in endotoxemia colon, which were effectively reversed by the pretreatment of BRD4 specific inhibitor JQ1 (**Figures 5A, B** **)**. These results disclosed BRD4 inhibition might prevent endotoxemia-induced colon damage through blocking inflammation-related pyroptosis.

## Discussion

Endotoxemia is a severe inflammation response induced by infection (35). The clinical practice is mainly focused on organ systems such as liver, lung, thymus, spleen, and so on, but the evaluation and monitor on endotoxemia-induced gut especially colon dysfunction are less accessible. In this study, we confirmed the destroyed colon mucosal integrity with crypt atrophy and goblet cell reduction in LPS-induced endotoxemia mice model. Moreover, downregulated tight junction proteins occludin and ZO1 were also detected to further verify the colon barrier damage in endotoxemia. So, it is really pressing to find a potential therapeutic compound to treat endotoxemia-related colon injury.

BRD4 is just like a dancer on the stage of inflammation, and is functionally required for inflammatory gene regulatory network depended on NF κB signal (36). It was demonstrated that miRNA302e attenuated inflammation in infantile pneumonia through the RelA/BRD4/NF κB signaling pathway (37). In addition, infection of respiratory syncytial virus induced airway inflammation through the coupling between BRD4 and NF κB (38). In this paper, we explored the functions of BRD4 on inflammation of endotoxemia colon. In consistent with the study above, we found elevation of pro-inflammation cytokines IL6, IL18, and IL1β in colon of mice suffering the inflammation-related disease endotoxemia, and BRD4 and phosphorylated NF κB also presented higher expression levels in endotoxemia colon compared to the control group. However, pretreatment with the BRD4 inhibitor JQ1 prevented the upregulated levels of inflammation cytokine and phosphorylated NF κB. Especially, JQ1 further rescued the missing expression of tight junction proteins occludin and ZO1, and obviously reversed the colon integrity in endotoxemia. These results above powerfully explicated BRD4 inhibition by JQ1 effectively protected colon from endotoxemia-induced inflammation injury *via* NF κB signal.

NF κB signal was reported to modulate NLRP3 inflammasome-induced pyroptosis in adipose inflammation (39) and inflammatory bowel disease (40). Since pyroptosis is a novel proinflammatory form of cell death that often occurred in inflammatory pathogenesis, we next confirmed the pyroptosis in endotoxemia colon, and interestingly discovered the activation of NLRP3/ASC/caspase 1 complex and even the elevation of pyroptosis markers—gasdermins family such as GSDMD, GSDME, and GSDMA. These results reminded us that inflammatory injury in endotoxemia colon could be a direct result of pyroptosis. As we described in the introduction part, BRD4 was only reported to affect pyroptosis in cerebral ischemia and renal cancer. But it is still unclear whether there is a link between BRD4 and pyroptosis in endotoxemia colon. In this study, JQ1 pretreatment inactivated the NLRP3/ASC/caspase 1 inflammasome assembly, and eliminated the elevation of gasdermins, which indicated BRD4 might promoted pyroptosis to lead endotoxemia colon injury.

In this paper, we mainly focused on affirming the connection between BRD4 and pyroptosis in endotoxemia colon. Since the mature study on the mechanism between BRD4 and NF κB signal in inflammation response have been carried out well as described before, we did not repeat the confirmatory experiments in this study. As shown in **Figure S1**, gene markers of multiple colon cells showed alteration, including Paneth cells, goblet cells, stem cells, lymphocytes, T cells, and microphages. Except Paneth cells, levels of most cell types are altered, which indicated there was a complex response system in endotoxemia colon. But it is still unclear how these epithelial cells and immune/inflammatory cells participate in the colonic injury induced by LPS. We will further investigate the details in the future.

## Conclusion

In summary, we found the elevated expression of BRD4 in damaged endotoxemia colon with tight junction injury, and further used JQ1 as a BRD4 inhibitor to verify that the inflammatory pyroptosis was induced by BRD4 *via* NF κB signal in endotoxemia colon.

## Data Availability Statement

The raw data supporting the conclusions of this article will be made available by the authors, without undue reservation.

## Ethics Statement

The animal study was reviewed and approved by the Institutional Animal Care and Use Committee at the First Affiliated Hospital of University of South China.

## Author Contributions

LC, XLZ, and WC conceived and designed the project, wrote the manuscript, and approved the final manuscript. LC, XLZ, MM, WL, and HY extracted and analyzed data, wrote the part of the results and discussion of the manuscript. WC, ML, MS, YZ, and YD conducted the experiments and revised the manuscript. JL and XYZ designed the project, and approved the final version. All authors contributed to the article and approved the submitted version. 

## Funding

This work was supported by the Project of Hunan Health Committee (202110002229) and Natural Science Foundation of Hunan Province (2019JJ50545), and the National Natural Science Foundation of China (81873651, 81901147).

## Conflict of Interest

The authors declare that the research was conducted in the absence of any commercial or financial relationships that could be construed as a potential conflict of interest.

## Supplementary Material

Supplementary Figure 1 Levels of gene markers of multiple cell-types in colon detected by Q-PCR.

Supplementary Figure 2 Immunofluorescence co-localization of BRD4 and TJ protein occludin in colon.

Supplementary Figure 3 Gene levels of anti-inflammation factors were upregulated by JQ1.

Supplementary Figure 4 TUNEL assay showed that JQ1 also blocked apoptosis of colon cells in endotoxemia.

Supplementary Table 1 Supplemental information of primer sequences.
