# Supplementary figures and images for: JQ1 as a BRD4 Inhibitor Blocks Inflammatory Pyroptosis-Related Acute Colon Injury Induced by LPS
Source: Front Immunol. 2021 Feb 18;12:609319. doi: 10.3389/fimmu.2021.609319 (PMC7930386; doi:10.3389/fimmu.2021.609319)

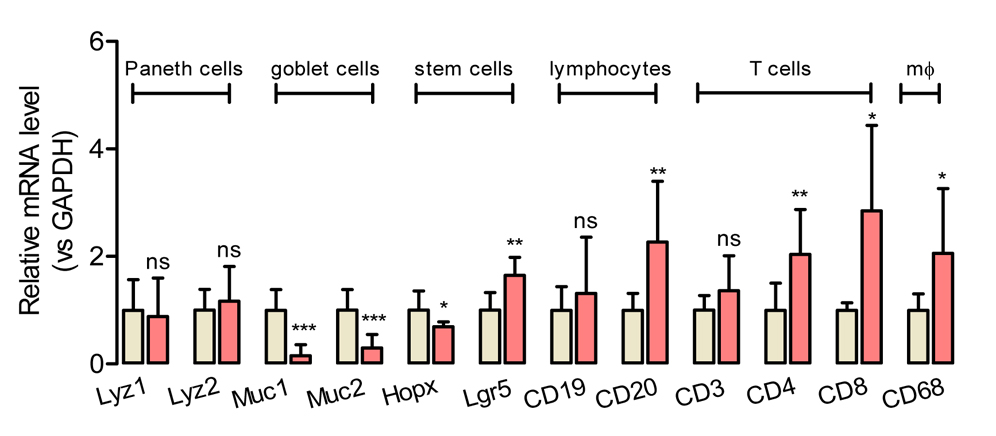

Supplement: Supplementary Figure 1 — Levels of gene markers of multiple cell-types in colon detected by Q-PCR. [file Image_1.jpg]

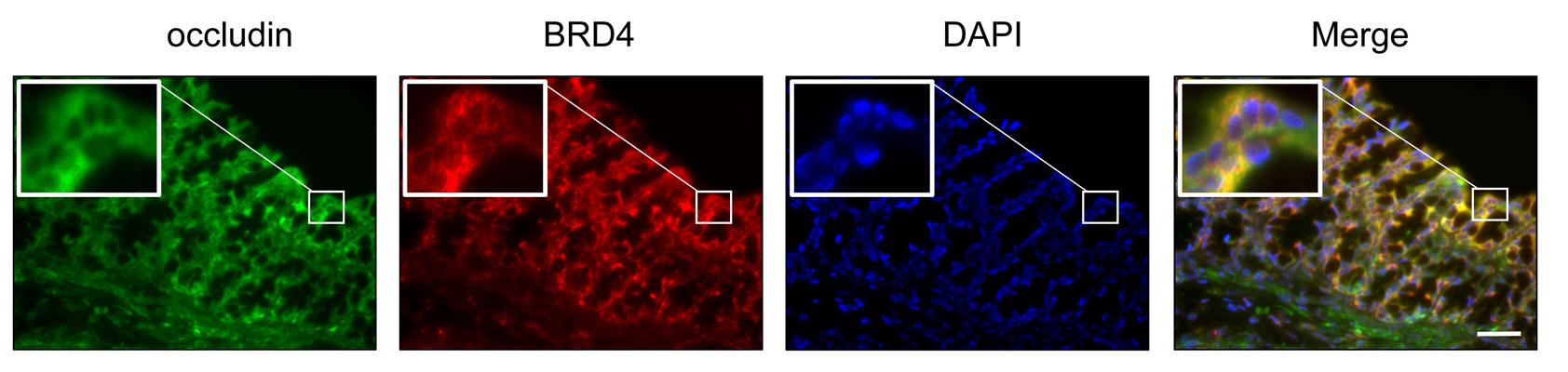

Supplement: Supplementary Figure 2 — Immunofluorescence co-localization of BRD4 and TJ protein occludin in colon. [file Image_2.jpg]

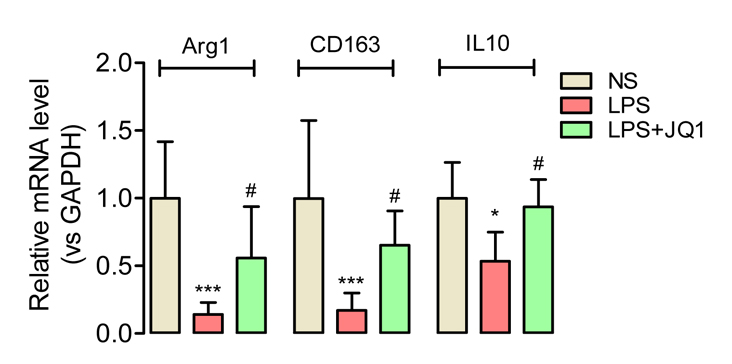

Supplement: Supplementary Figure 3 — Gene levels of anti-inflammation factors were upregulated by JQ1. [file Image_3.jpg]

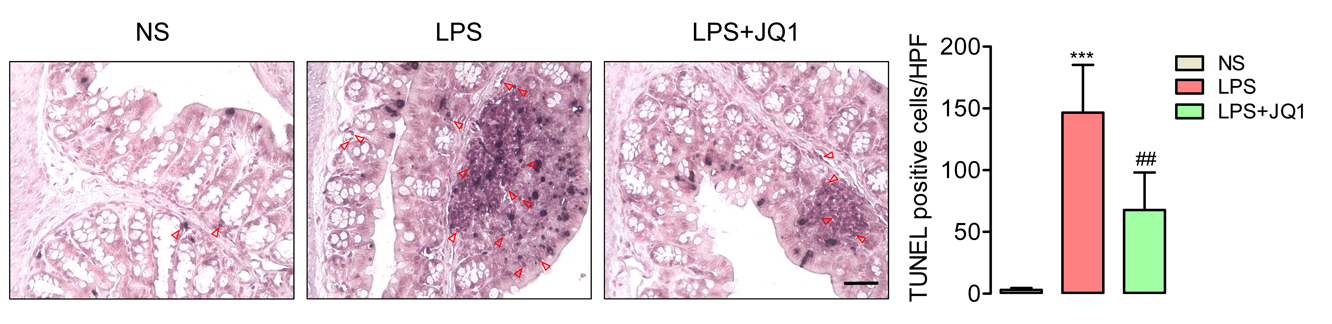

Supplement: Supplementary Figure 4 — TUNEL assay showed that JQ1 also blocked apoptosis of colon cells in endotoxemia. [file Image_4.jpg]
