# Supplementary material for: JQ1 as a BRD4 Inhibitor Blocks Inflammatory Pyroptosis-Related Acute Colon Injury Induced by LPS
Source: Front Immunol. 2021 Feb 18;12:609319. doi: 10.3389/fimmu.2021.609319 (PMC7930386; doi:10.3389/fimmu.2021.609319)
Supplement: Supplementary Table 1 — Supplemental information of primer sequences. [file Table_1.doc]

**Table S1.** Supplemental information of primer sequences.

| Gene | Sense | Anti-sense |
| --- | --- | --- |
| Lyz1 | AGATCAATAGCCGATACTGGTG | TCTCGGTTTTGACATTGTGTTC |
| Lyz2 | AGGTCTATGAACGTTGTGAGTT | ACCAGTATCGGCTATTGATCTG |
| Muc1 | ACGTGAAGTCACAGCTTATACA | AGGGCAAGGAAATAGACGATAG |
| Muc2 | TGCTGACGAGTGGTTGGTGAATG | TGATGAGGTGGCAGACAGGAGAC |
| Hopx | TGGAGTACAACTTCAACAAGGT | CTAGTCCGTAACAGATCTGCAT |
| Lgr5 | CGCAGCCTAAACCTGAGTTATA | CATTGCTGTTGAGGTACACTTC |
| CD19 | GTGCATCCTAGGAGACCTAATG | CAGGTTTCCTCGGAGACAATAA |
| CD20 | TCCCCATCTACACAGTACTGTA | GGGAAGATACTCCACTTAGCTC |
| CD3 | CATCTCAGGAACCAGTGTAGAG | CTCGAGCTTTCAGGTACAAGTA |
| CD4 | CAGCATGGCAAAGGTGTATTAA | GACTGAAGGTCACTTTGAACAC |
| CD8 | GTCTATATGGCTTCATCCCACA | GTTCAGGGTGAGAACGTACTTA |
| CD68 | GAAATGTCACAGTTCACACCAG | GGATCTTGGACTAGTAGCAGTG |
| Arg1 | CATATCTGCCAAAGACATCGTG | GACATCAAAGCTCAGGTGAATC |
| IL8 | CTGTTGGCCCAATTACTAACAG | TCCCGAATTGGAAAGGGAAATA |
| CD163 | GTTTGTGGAGCCATTCTATTGG | GGAAACTGTAAGTCGCTGAATC |
